# BioViz *Connect*: Web Application Linking CyVerse Cloud Resources to Genomic Visualization in the Integrated Genome Browser

**DOI:** 10.3389/fbinf.2022.764619

**Published:** 2022-05-23

**Authors:** Karthik Raveendran, Nowlan H. Freese, Chaitanya Kintali, Srishti Tiwari, Pawan Bole, Chester Dias, Ann E. Loraine

**Affiliations:** Department of Bioinformatics and Genomics, University of North Carolina at Charlotte, Charlotte, NC, United States

**Keywords:** SR45a, AT1G07350, Arabidopsis, abiotic stress, Integrated Genome Browser, CyVerse, visualization, Terrain API

## Abstract

Genomics researchers do better work when they can interactively explore and visualize data. Due to the vast size of experimental datasets, researchers are increasingly using powerful, cloud-based systems to process and analyze data. These remote systems, called science gateways, offer user-friendly, Web-based access to high performance computing and storage resources, but typically lack interactive visualization capability. In this paper, we present BioViz *Connect*, a middleware Web application that links CyVerse science gateway resources to the Integrated Genome Browser (IGB), a highly interactive native application implemented in Java that runs on the user’s personal computer. Using BioViz *Connect*, users can 1) stream data from the CyVerse data store into IGB for visualization, 2) improve the IGB user experience for themselves and others by adding IGB specific metadata to CyVerse data files, including genome version and track appearance, and 3) run compute-intensive visual analytics functions on CyVerse infrastructure to create new datasets for visualization in IGB or other applications. To demonstrate how BioViz *Connect* facilitates interactive data visualization, we describe an example RNA-Seq data analysis investigating how heat and desiccation stresses affect gene expression in the model plant *Arabidopsis thaliana*. The RNA-Seq use case illustrates how interactive visualization with IGB can help a user identify problematic experimental samples, sanity-check results using a positive control, and create new data files for interactive visualization in IGB (or other tools) using a Docker image deployed to CyVerse *via* the Terrain API. Lastly, we discuss limitations of the technologies used and suggest opportunities for future work. BioViz *Connect* is available from https://bioviz.org.

## Introduction

Science gateways are Web sites that implement user-friendly interfaces to high performance computing and storage systems (Wilkins-Diehr et al., 2008). Science gateways typically assemble and curate discipline-specific, command-line, Unix-based tools within a single, easy-to-use interface, enabling users to run compute-intensive processing on datasets too large for a personal computer (Giardine et al., 2005; Goff et al., 2011; Merchant et al., 2016). In a typical use case, domain researchers upload their “raw” (unprocessed) data to the gateway site and then operate the gateway’s Web-based interface to create custom processing and analysis pipelines, where a pipeline is defined as tasks performed in sequence by non-interactive tools which emit and consume well-understood file types and formats. Common pipeline tasks in genomics include aligning RNA-Seq sequences onto a reference genome to produce BAM (binary alignment) format files (Li et al., 2009), generating scaled RNA-Seq coverage graphs from the “BAM” files using tools such as deepTools bamCoverage (Ramirez et al., 2016), or searching promoter regions for sequence motifs common to sets of similarly regulated genes using tools such as DREME (Bailey, 2011).

A science gateway aims to provide a single point of access for tools needed to process and analyze data from a research project. However, native visualization tools with their own graphical user interfaces separate from a Web browser are difficult to use with Web-based science gateway systems. The Integrated Genome Browser from BioViz.org (Nicol et al., 2009; Freese et al., 2016) and the Broad Institute’s Integrative Genomics Viewer (Robinson et al., 2011) exemplify this problem. Both tools require that data files reside on the user’s local file system or that they be accessible *via* HTTP (hypertext transfer protocol) and addressable *via* a file-specific URL (Uniform Resource Locator). If the gateway system does not allow URL-based access to data, then users must download the data files onto their local computer file system, which may not be practical or allowed.

Related problems confront visualization systems implemented as Web applications, deployed on Web hosts and not the user’s local computer. Using Web applications to visualize data can be even more challenging for users, because these applications often require hard-to-set-up data storage and delivery mechanisms specialized to the application. To view one’s data using the Web-based UCSC Genome Browser software, for example, users can either deploy their own copy of the software, which is difficult, or they can instead set up a UCSC Track Hub server, which is less technically challenging but nonetheless requires Track Hub-specific meta-data files to be created and configured (Raney et al., 2014). Similarly, using the JBrowse Web-based genome browser requires deploying data in JBrowse-compatible formats (Buels et al., 2016).

Another typical requirement for science gateways is extensibility, meaning they require a way for gateway developers or users to add new tools to the system to accommodate or even potentiate new directions for research. The CyVerse science gateway, the focus of this article, supports extensibility by allowing developers to create and deploy CyVerse Apps, which are user-contributed container images that run within a CyVerse-provided container environment (Devisetty et al., 2016). Users create containers using Docker and then contribute their container image along with metadata specifying input parameters and accepted data types to CyVerse. Once accepted and deployed, the container is configured to run as an asynchronous “job” within the CyVerse infrastructure *via* a queuing system. Thus, Apps run non-interactively and therefore are not well-suited to providing interactive, exploratory visualization. However, these Apps do provide a means to create new input data for visualization, as we explore here.

In this paper, we introduce BioViz *Connect*, a Web application that overcomes limitations described above to add genome visualization capability to the CyVerse science gateway system. Previously called iPlant, the CyVerse science gateway is a United States National Science Foundation funded cyberinfrastructure project with the aim of providing computational resources for life sciences researchers (Goff et al., 2011; Merchant et al., 2016). We chose to work with CyVerse in this study because it features a rich Application Programming Interface (API), the Terrain REST API, that supports secure computational access to CyVerse data storage and analysis resources.

Using this API, we implemented a new visualization-focused interface to these resources, called BioViz *Connect*, using the Integrated Genome Browser (IGB) as the demonstration application. We selected IGB because it offers one of the richest feature sets for visual analysis in genomics [for descriptions of IGB functionality, see (Nicol et al., 2009; Gulledge et al., 2014; Loraine et al., 2015; Freese et al., 2016; Mall et al., 2016)] and because we are members of the core IGB development team. Therefore, we possessed insider’s knowledge of the featured visualization application that allowed us to modify IGB as needed for the project.

BioViz *Connect* enables users of Integrated Genome Browser to visually analyze their CyVerse data without having to download entire files to their local computer or migrate their data into application specific data stores. BioViz *Connect* lets users annotate their data sets with metadata, which control how the data will look when imported into the IGB and also indicate the genome version referenced in the data. Finally, BioViz *Connect* lets users run compute-intensive visual analytics algorithms, implemented as CyVerse Apps.

In the following sections, we describe how BioViz *Connect* is implemented, explaining the technology stack used and how BioViz *Connect* interacts with the CyVerse science gateway resources *via* its Terrain API. Next, we describe how BioViz *Connect* enables flow of data into the IGB desktop software by activating a REST API endpoint residing in IGB itself. To illustrate the functionality, we describe an example use case scenario for BioViz *Connect* in which a hypothetical analyst uses visualization and visual analytics tools within IGB in conjunction with their CyVerse account to quality-check and analyze an RNA-Seq data set from *Arabidopsis thaliana* plants undergoing desiccation and heat stresses. Lastly, we discuss insights gained from implementing BioViz *Connect*, describe limitations of the technology used, and propose how these limitations might be overcome. BioViz *Connect* represents a next step toward building integrated, user-friendly computational environments that blend powerful local tools like IGB with even more powerful remote infrastructures like CyVerse, creating new possibilities for users to discover biologically meaningful features in data while avoiding artifacts.

## Design and Implementation Details

In the following sections, we describe technical aspects of how BioViz *Connect* is implemented, while also describing user interface design choices intended to improve both usability and transparency for users.

### BioViz *Connect* Client and Server-Side Design

**FIGURE 1:**
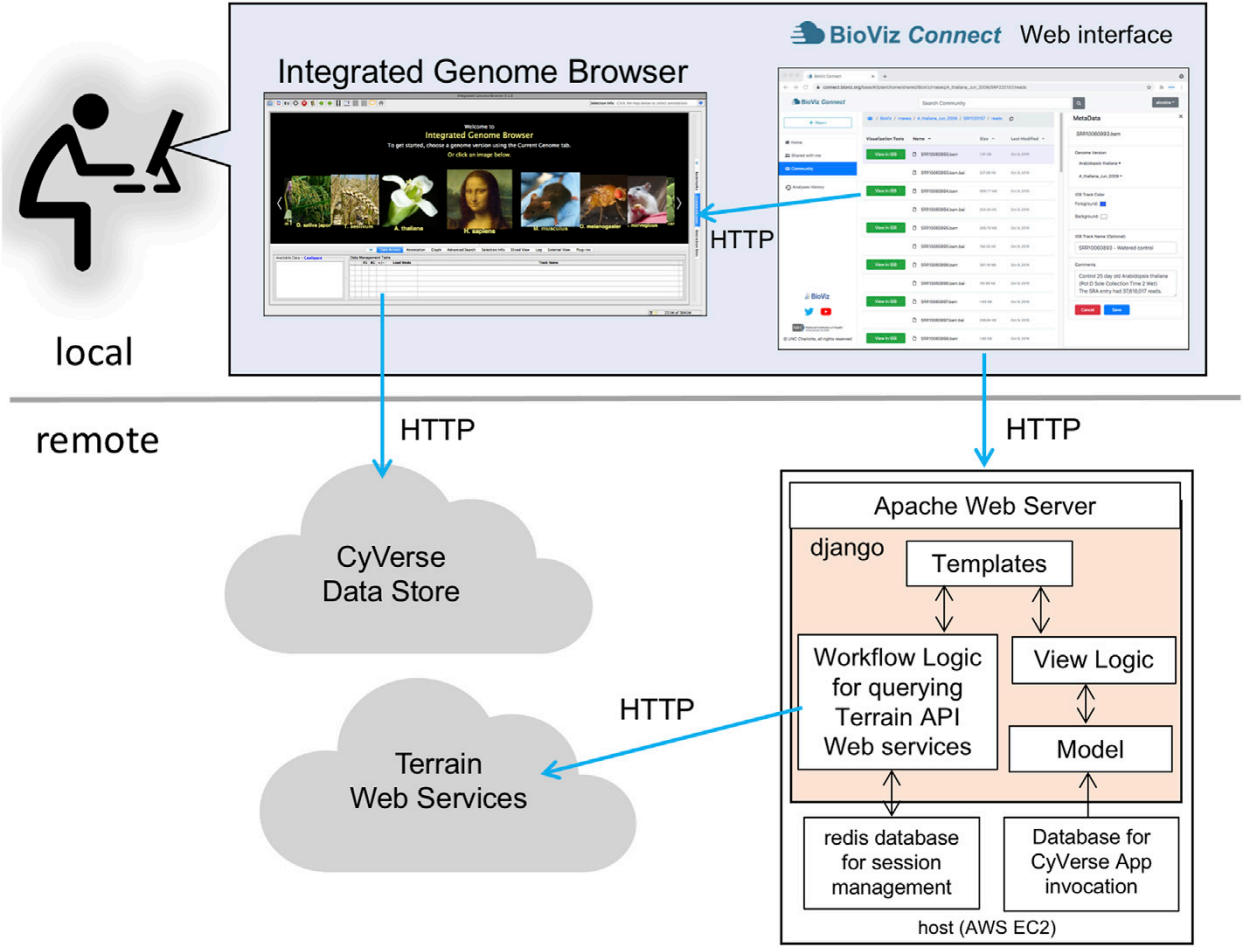
Diagram illustrating local client and remote server-side design of BioViz *Connect*. After users log in using their CyVerse user credentials, the BioViz *Connect* Web interface appears in their Web browser. Clicking the “View in IGB” button causes the browser to make HTTP requests to a localhost REST endpoint within IGB. These HTTP requests include information IGB uses to issue new HTTP requests for data resources. The BioViz *Connect* Web interface is populated *via* HTTP requests made to a remote host running the Apache Web server, which forwards these requests (using a reverse proxy mechanism) to the BioViz *Connect* Web application, implemented using the Django Web application framework. BioViz *Connect* then translates and forwards these user requests *via* HTTP to REST API endpoints that are part of the Terrain API, which manages interactions with the CyVerse cloud.

BioViz *Connect* consists of two parts: a JavaScript-based user interface that runs in a Web browser and a server-side application that manages authentication and communication with Terrain API endpoints. The user interface code on the client-side is implemented using HTML5, CSS, Bootstrap 4.3.1, JavaScript, and jQuery 1.10.2. The server-side code is implemented in python3 using the Django web application framework (Figure 1). The currently available production instance of BioViz *Connect* is deployed on an Ubuntu 18.04 system and hosted using the apache2 Web server software as a reverse proxy. BioViz *Connect* code is open source and available from https://bitbucket.org/lorainelab/bioviz-connect.

### User and Password Management

To use BioViz *Connect*, users must first obtain a CyVerse Discovery Environment account by registering at https://user.cyverse.org/register (CyVerse, RRID: SCR_014531). At the time of this writing, there is no charge for this account. BioViz *Connect* delegates user management, including logging in and password management, to the Central Authentication Service (CAS) OAuth service hosted and maintained by CyVerse (Figure 1). Thus, CyVerse infrastructure manages user accounts and information; no BioViz-specific accounts or passwords are required, and the BioViz *Connect* software never gains access to the user’s CyVerse password. After a user has logged in to BioViz *Connect* using their CyVerse credentials, BioViz *Connect* uses its server-side Redis database to store a user-specific access token for the duration of the session, a token that allows BioViz *Connect* to access and modify user data stored in CyVerse infrastructure *via* the Terrain API on the user’s behalf.

### Integrated Genome Browser REST Endpoint

Integrated Genome Browser is a free, open-source desktop software program written in Java which users download and install on their local computer systems (IGB, RRID: SCR_011792) (Nicol et al., 2009; Freese et al., 2016). Installers for Linux, MacOS, and Windows platforms are available at https://bioviz.org.

The IGB source code resides in a git repository hosted on Atlassian’s bitbucket.org site (https://bitbucket.org/lorainelab/integrated-genome-browser). When viewed on the BitBucket git repository’s Web site, changes to the code called “commits” link to pages on the project management Web site documenting the motivation for the change and/or technical challenges encountered, thus making the source code easier to manage and understand. The project management Web site uses Jira from Atlassian Software, with URL https://jira.bioviz.org. IGB version 9.1.4 or greater is required for IGB to connect to BioViz *Connect*.

IGB contains a simple Web server configured to respond to REST-style queries on an IGB-specific port on the user’s local computer. JavaScript code downloaded into the Web browser when users visit BioViz *Connect* pages enables requesting URLs addressed to “localhost”, the user’s computer, using the IGB-specific port. IGB intercepts these requests and performs actions dictated by parameters embedded in the URL text. This mechanism repurposes a REST endpoint dating from the earliest releases of IGB from the early 2000s. The IGB Users’ Guide hosted at https://wiki.bioviz.org/confluence describes these and other features.

### BioViz *Connect* Metadata

BioViz *Connect* uses the Terrain Metadata API to manage and obtain IGB-specific metadata for files and folders. The Terrain API represents metadata items as triplets containing Attribute, Value, and Unit. A metadata item’s Attribute attaches meaning to what the metadata contains, and application developers can create their own custom Attributes to support diverse purposes. For example, since BioViz *Connect* is concerned with genomic data visualization, we created custom Attributes signaling genome assembly version, visual style information such as foreground color and background color, and free text comments on the data provided by the user, which are displayed in BioViz *Connect*’s Web interface. A metadata item’s Value is specific to the file or folder being tagged. BioViz *Connect* uses the Unit value to indicate that the metadata element concerns IGB and the BioViz *Connect* application.

The genome identifier attribute requires further explanation, as matching genome version names across systems has caused many problems for genome browsers and their users. Integrated Genome Browser, like many other systems, uses an application-specific scheme for naming genome versions, and contains a listing of synonyms matching these IGB-specific names onto genome version names from other systems. For example, the IGB genome version named H_sapiens_Feb_2009 is the same as UCSC genome version name hg17, which is the same as NCBI version 35. The BioViz *Connect* user interface includes components for users to view, designate, or change the genome version metadata associated with individual files. To ensure compatibility with IGB, BioViz *Connect* uses a list of IGB-formatted genome identifiers hosted on the IGB Quickload site (http://igbquickload.org/quickload/) to configure the genome version selection components, implemented as menus. When users operate the interface to view data within IGB, the genome version metadata, along with style metadata, are passed to IGB *via* its localhost REST endpoint. This ensures that the data appear in the context of the correct genome assembly, alongside other data already loaded from BioViz *Connect* or other sources, while also enabling the user to specify in advance how the data will look once it appears in IGB. In addition, if other users load the same files, the data will look the same.

### Enabling Access to Data *via* Public URLs

The flow of data from CyVerse into IGB depends on two key technical features of the CyVerse data storage and hosting system. First, the Terrain API enables users to create publicly accessible URLs for data files in their accounts, and these URLs can be enabled or disabled at will. In the current implementation, URLs created in this way are accessible to any internet user. Second, the CyVerse infrastructure supports HTTP range requests for these URLs, enabling clients such as IGB to request subsets of data, thus avoiding having to download or transfer an entire data file.

**FIGURE 2:**
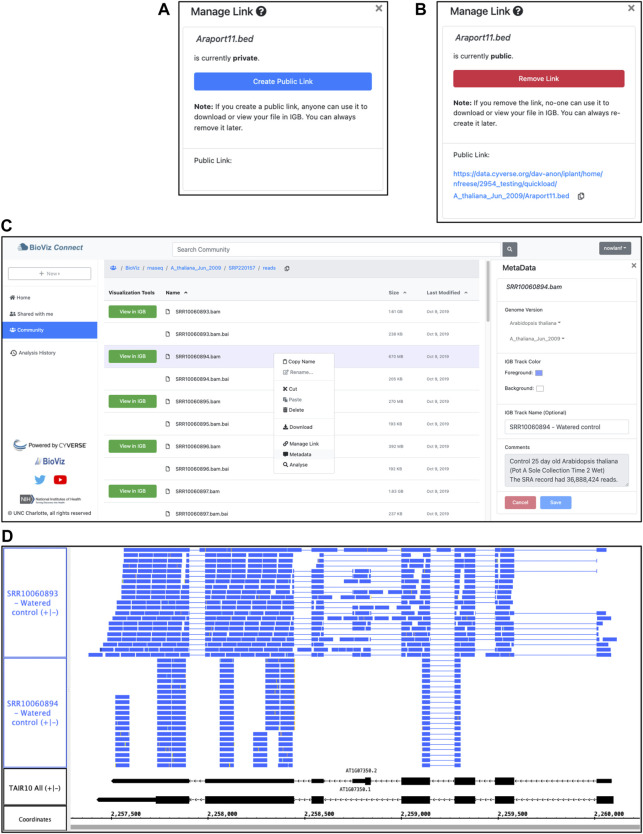
BioViz *Connect* interface and IGB data visualization. **(A,B)** BioViz *Connect* “Manage Link” interface, from the right panel display. By default, files are not publicly accessible, and the interface appears as in **(A)**. Clicking the button labeled “Create Public Link” creates a public link, switching the display to the image shown in **(B)**. **(C)** BioViz *Connect* main page. The left panel shows shortcuts to home, shared, community folders. The middle panel lists files and folders. The right panel shows the selected file’s metadata. **(D)** SRR10060893.bam and SRR10060894.bam files viewed in IGB overlapping the SR45a gene of *A. thaliana*. The track labeled TAIR10 mRNA shows SR45a gene models AT1G07350.1 and AT1G07350.2.

The BioViz *Connect* interface is designed to make the process of managing these URLs as easy as possible, similar to commercial cloud storage systems such as Dropbox and Google Drive that let users create, destroy, and manage public links to individual files and folders. Within the BioViz *Connect* interface, users create URLs for individual files by right clicking the file and selecting the “Manage Link” option. Selecting this option opens a right panel display in which the current status of the file is shown, and users can toggle between making the file public or private (Figures 2A,B).

As shown in Figure 2B, the text of this public URL is visible to the user, and users can copy it to their system clipboard by clicking the “copy” icon. The Terrain API determines the link text, and currently, it always contains the user’s chosen name for the file and the path to the file within the virtual file system, preceded by the prefix shown in Figure 2B. We expose this detail to users because increasingly many researchers are using their CyVerse accounts to host files, and the current transparency and predictability of these URLs seems important for them to know about. Likewise, if the pattern ever changes, they will need to know this, as well.

### BioViz *Connect* Deployment

BioViz *Connect* is managed using ansible roles and playbooks publicly available in a git repository from https://bitbucket.org/lorainelab/bioviz-connect-playbooks. The playbooks contain two sets of tasks. One set of tasks creates a virtual machine using the Amazon EC2 Web service. Once the host is created and running, a second set of ansible tasks installs and configures software on the host, including an Apache2 Web server, a MySQL database, and the BioViz *Connect* code base. Playbook users can specify the BioViz *Connect* repository and branch they wish to deploy, which facilitates rapid testing of proposed new code. During the provisioning process, a call is made to a Terrain endpoint that provides a list of all CyVerse asynchronous analysis apps that can produce output visible to IGB. These data are then used to construct the “analysis” sections of the user interface, and are stored in the BioViz *Connect* relational database, co-located on the same host.

### BioViz *Connect* Interface for Running Visual Analysis Apps

When users right-click a file name in BioViz *Connect*, a context menu appears with an option labeled “Analyse.” Information about IGB-compatible Apps, the file types they can accept, and App parameters are stored in the relational database configured during deployment as described above. When a user selects this option, BioViz *Connect* queries the database to identify IGB Community Apps that accept the file as input, and these are then displayed to the user. Once the user has selected an App, another query retrieves additional information about it, such as user-friendly description of what the App does, which is then displayed to the user.

The CyVerse ecosystem contains many hundreds of Apps, many of which are redundant or obsolete, and so the BioViz Team controls which ones are shown to users by adding them to the IGB Community, a CyVerse organizing concept that groups resources (such as Apps) according to which users can use or modify them. BioViz *Connect* only shows Apps that have been added to the IGB Community.

### RNA-Seq Data

RNA-Seq data presented in the use case scenario are from Sequence Read Archive Bioproject PRJNA509437 (Leinonen et al., 2011), an experiment in which Arabidopsis plants underwent either a 3-h, non-lethal heat stress or a multi-day desiccation stress. Two post-treatment sample time points were collected for treated plants and their untreated control counterparts, with two to four replicates per sample type and 23 samples in total. Sample libraries were sequenced in single-end runs of the Illumina platform and are identified by their run identifiers. BAM files were generated by aligning sequence reads to the Arabidopsis June 2009 reference genome assembly using TopHat2 (TopHat, RRID: SCR_013035) (Kim et al., 2013). The data are available in the Community folder of publicly accessible datasets, represented as a folder in the left-side panel of the BioViz *Connect* display.

## Results

### Understanding and Navigating the BioViz *Connect* Interface

Our design goal in creating BioViz *Connect* was to give the user a feeling of almost limitless computational power and space by integrating seamlessly with the CyVerse “cloud.” Doing so requires that users identify themselves to the system by entering a username and password, but how this process takes place can easily destroy the illusion of seamless access. To avoid this, we used an OAUTH-style Terrain API endpoint that delegates logins to CyVerse infrastructure, preventing BioViz *Connect* from learning the user’s password.

To begin a session with BioViz *Connect*, the user opens the BioViz.org website in a Web browser, selects the link labeled BioViz *Connect*, and then clicks the link labeled “Sign in with your CyVerse ID”. This action opens a Central Authentication Service (CAS) page, hosted by CyVerse, where users enter their CyVerse username and password, or sign up for a new account if they do not already have one. The Web browser then returns to a “call-back” URL on the BioViz.org site, which displays the BioViz *Connect* user interface, a browsable, sortable, paginated view of the user’s CyVerse home directory and its contents (Figure 2C).

This view of files and data resembles the interface for commercial, consumer-focused cloud storage systems, a deliberate design choice aimed at building on many users’ familiarity with Google Drive, the Dropbox Web interface, and others. This interface displays a sortable, table-based view of the user’s home directory within the CyVerse file storage system, displaying a listing of files and folders the user has uploaded to their account or created using CyVerse Apps, including BioViz *Connect* Apps described in later sections. Single-clicking a file or folder selects it, double-clicking a folder opens it and displays the contents, and double-clicking a file opens a metadata display showing information about the file (Figure 2C). A bread crumb display at the top of the page shows the path from the root folder to the currently opened folder, and a copy icon next to the breadcrumb allows the user to copy the folder name and path. The browser forward and back buttons work as expected, and users can bookmark individual screens for faster navigation. The URLs displayed in the browser’s URL bar match the currently opened folder’s location, making the interface feel more polished and user-friendly by ensuring that every user-facing detail, including the URL, mimic and reinforce how the user has organized their data within the CyVerse virtual file system.

The top part of every BioViz *Connect* page also features a search bar that can be used to find files and folders with names matching a user-entered query string. Matches are returned in a list view similar to the original table view, and users can sort the results list by name, size, or date modified. Only files for which the user has read access and that reside in the currently visible section (Home, Community, or Shared with me) are returned. On the left side of every page, BioViz *Connect* displays icons representing shortcut links to the user’s home directory, a publicly available community data folder, and other destinations. The “Community” folder contains data published for all CyVerse users, including the example RNA-Seq data set for the use case scenario described in the next section.

### Using BioViz *Connect* to View Data in Integrated Genome Browser

To demonstrate BioViz *Connect* functionality, we next describe an example use case scenario in which a hypothetical researcher visually analyzes data from a typical RNA-Seq experiment. The use case focuses on two main tasks: visually checking data quality and then confirming differential expression of a control gene known to be regulated by the treatment.

The experimental design included two treatments, heat and desiccation stress, their controls, and two time points, totaling six sample types, each with two to four replicates. The RNA-Seq sequences are available in the Sequence Read Archive, and the researcher has obtained the data, aligned it to the reference genome, and then contributed the files to the Community folder. Alignment files are stored in the file path “*BioViz/rnaseq/A_thaliana_Jun_2009/SRP220157/reads*”. The user has also annotated each file using the BioViz *Connect* interface, adding the genome version, visual style information, and notes describing each sample.

Now that the data are organized and annotated, the researcher uses the BioViz *Connect* interface to import the data into Integrated Genome Browser for visualization and proceeds to look at each file, one by one, to check the quality of the alignments and confirm file identity. BioViz *Connect* makes this task easy to perform. To illustrate, we discuss RNA-Seq alignment files SRR10060893.bam and SRR10060894.bam, replicate control samples from time point one of the heat stress treatment. A quick scan of files listed in the BioViz *Connect* table view shows that SRR10060893.bam has size 1.61 GB, about twice the size of SRR10060894.bam, which is 0.669 GB. The user has annotated the files with the number of sequence reads obtained per sample, around 37 million for each. Because the samples were sequenced to about the same depth, their resulting alignment files ought to have similar sizes. Visualizing the sequence read alignments will help explain the discrepancy.

To visualize the alignments, the user launches Integrated Genome Browser, which is already installed on the local computer, downloaded from the BioViz.org Web site. Once IGB is running, the user clicks the “View in IGB” button available in the “Visualization Tools” column in the BioViz *Connect* table view, repeating this action for each file (Figure 2C). This action causes JavaScript code running within the Web browser to request data from a local URL (domain “localhost”) corresponding to a REST endpoint implemented within IGB. The URL includes parameters such as the publicly accessible URL for the data file, the IGB name of its reference genome, and visual style information indicating how the file should look once loaded into IGB. In response, IGB opens the requested genome version associated with the file and adds the file as a new track to the display.

To check assumptions about a new data set, it is useful to visualize a gene of known behavior, such as a gene already known to be regulated by the experimental treatment. Prior work from our lab and others have shown that SR45a, encoding an RNA-binding protein, is upregulated by heat and desiccation stresses, making it a good choice for this purpose (Yoshimura et al., 2011; Gulledge et al., 2012). To find the gene, the analyst enters SR45a into IGB’s search interface at the top left of the IGB window, which zooms and pans the display to the gene’s position in the genome. Next, the user loads the alignments into the display by clicking the “Load Data” button at the top right of the IGB window. Once the data load, the user customizes track appearance by modifying vertical zoom setting and changing the number of sequences that can be shown individually in a track (stack height), creating the view shown in Figure 2D.

This customized view makes problems with SRR10060894 obvious at a glance. The alignments for this sample appear to stack on top of each other in orderly, uniform towers covering only 30% of the gene’s exonic sequence. By contrast, the alignments for sample SRR10060893 cover most of the exonic sequence and also include many spliced reads split across introns. The sparser pattern observed in SRR10060894 typically arises when the library synthesis process included too many polymerase chain reaction amplification cycles, reducing the diversity of resulting sequence data. This pattern indicates that the user should exclude SRR10060894 from further analysis, but the other file appears to be fine.

### Comparing Sequencing Depth and Complexity Using Integrated Genome Browser Visual Analytics

**FIGURE 3:**
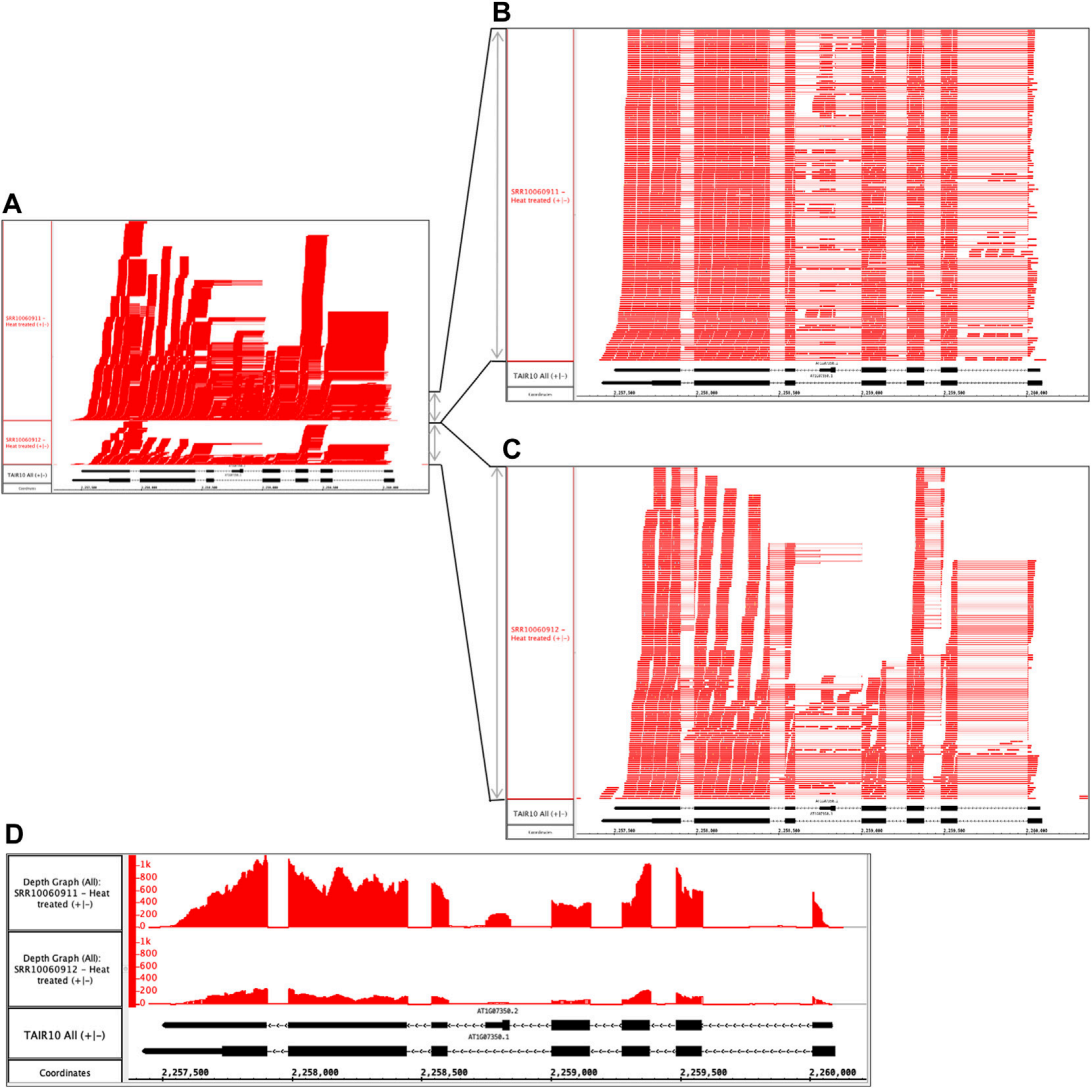
Heat treated samples viewed in IGB. **(A)** Vertical dimension is compressed to show all alignments. **(B)** SRR10060911 and **(C)** SRR10060912 tracks stretched vertically to reveal alignment patterns in more detail. **(D)** Alignment coverage graphs calculated within IGB using alignments from **(A)**. The y-axis values represent the number of aligned sequences per base pair position indicated on the coordinates track. The track labeled TAIR10 mRNA shows SR45a gene models AT1G07350.1 and AT1G07350.2.

Repeating the preceding process with other samples in the dataset, the user identifies another problematic pair of files. The files are replicates, but like the previous example, the files sizes differ. The alignments file SRR10060911.bam is 1.83 Gb, but its replicate SRR10060912.bam is only 0.454 Gb. Opening and viewing the alignment files in IGB, the user confirms that one file appears to contain more data than the other (Figure 3A). To quantify this observation, the user takes advantage of a simple, interactive visual analytics feature within IGB: selection-based counting. As with PowerPoint and many other graphical applications, IGB users can click-drag the mouse over graphical elements to select a group of items and then single-click while pressing SHIFT or CTRL-SHIFT keys to add or remove items from selection group. IGB reports the number of currently selected items in the Selection Info box at the top right of the IGB window. Using this feature, the researcher finds that sample SRR10060912 contains 1,925 alignments covering SR45a, and sample SRR10060911 has 10,867 alignments, nearly five times as many.

By further configuring track height and appearance settings, and operating IGB’s dynamic vertical and horizontal zoom controls, the user can stretch the display in each dimension independently to reveal more detail about the alignments (Figures 3B,C). From this new view of the data, the user can tentatively conclude that alignment pattern diversity is similar in each sample, but the depth of sequencing was greater in SRR10060911. To confirm the finding, the user then applies a visual analytics function (called a “Track Operation” within IGB) that creates coverage graphs, also called depth graphs, using data from the read alignment tracks (Figure 3D). To make a coverage graph, the user right-clicks a track label for a read alignment track and chooses option “Track Operations > Depth Graph (All).” This generates a new track showing a graph in which the y-axis indicates the number of sequences aligned per x-axis position, corresponding to base pair positions. After modifying the y-axis lower and upper boundary values (using controls in IGB’s Graph tab), the user again can observe that the pattern of alignments is similar between the two samples, but the overall level of sequencing was different. Thus, the file size difference most likely is due to a difference in sequencing depth rather than a problem with the library synthesis, as was the case in the previous example.

### Normalizing Coverage Graphs to Compare Gene Expression Visually

Coverage graphs set to the same scale allow comparing gene expression across sample types, but only if the libraries were sequenced to approximately the same depth. If not, then coverage graphs need to be normalized before comparing them. Scaling coverage graphs within IGB is impractical, however, as it would require downloading, reading, and processing the entire bam-format alignments file. A better approach is to off-load computationally intensive visual analytics tasks to CyVerse cloud computing resources. To demonstrate the value of this strategy, we deployed the deepTools genomeCoverage command line tool from the deepTools suite (Deeptools, RRID: SCR_016366) as a new IGB-friendly CyVerse App (Ramirez et al., 2016).

**FIGURE 4:**
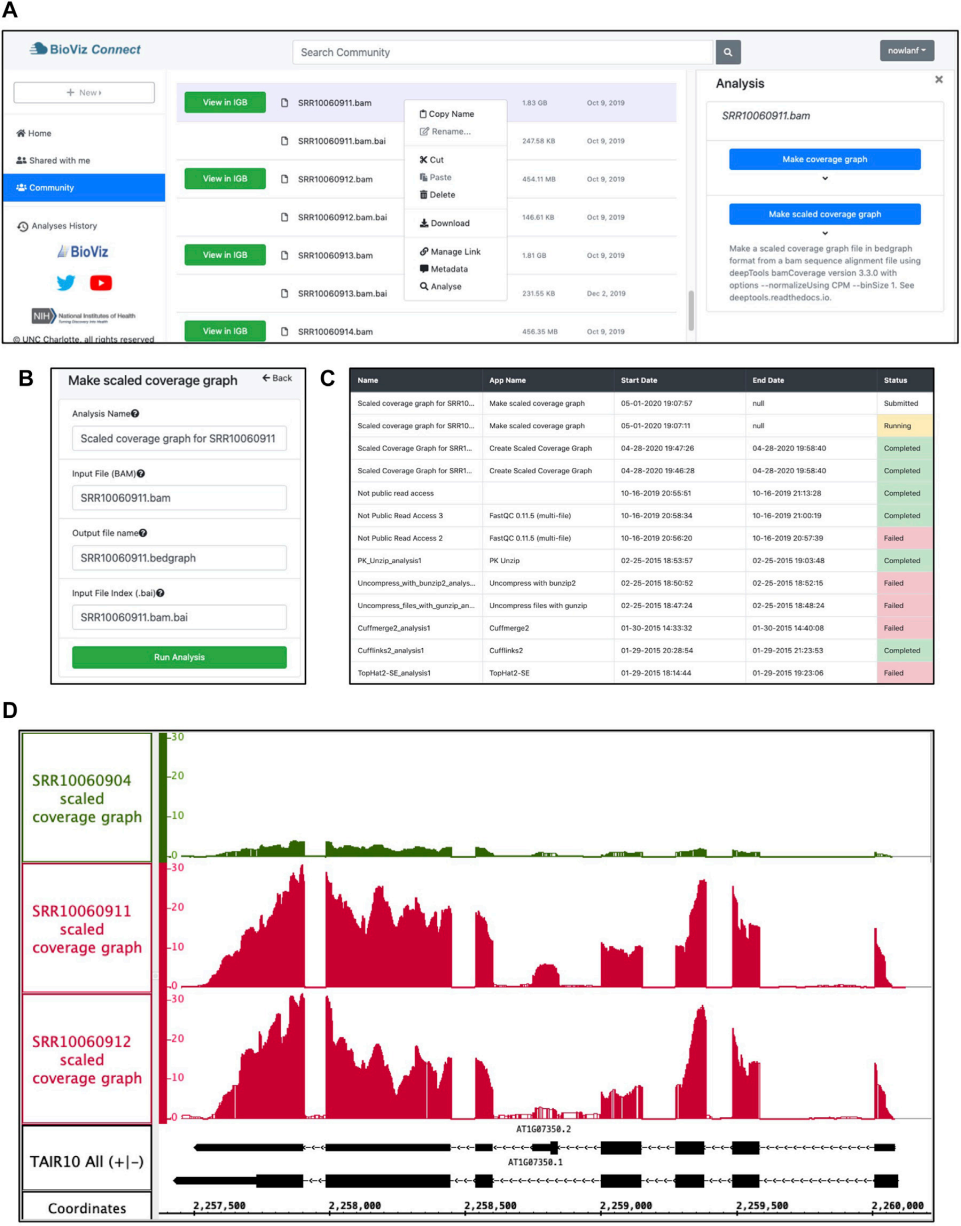
Example analysis in BioViz *Connect* with output visualized in IGB. **(A)** BioViz *Connect* main page with analysis right panel open. **(B)** Scaled coverage graph analysis options for naming the analysis, selecting input file, output file name, and index file selection. **(C)** Analyses History showing the status of current and previous jobs. **(D)** SRR10060904 (control), SRR10060911 (heat treated), and SRR10060912 (heat treated) scaled coverage graphs viewed in IGB overlapping the SR45a gene of *A. thaliana*.

To create a scaled coverage graph, the user returns to BioViz *Connect*, right-clicks a bam format file, and chooses “Analyse.” This opens the Analysis right-panel display, which lists all IGB-compatible CyVerse Apps that can accept the selected file type as input (Figure 4A). Selecting “Make scaled coverage graph” opens a form with options for creating the graph using the genomeCoverage algorithm (Figure 4B). The interface includes a place for the user to enter names for the analysis and for the output file that will be produced. The user then clicks “Run Analysis” button, which calls upon the CyVerse analysis API to run the App with specified parameters using CyVerse computing resources. The request to run the App and the work it performs are called “jobs,” and jobs are carried out asynchronously, running and completing only when resources they require become available, as with other systems set up for high-performance computing. Users can check job status by using the Analyses History in the BioViz *Connect* interface (Figure 4C), where Analyses are listed as Queued (waiting to run), Running, Failed, or Completed. The length of the time to complete a job is dependent on the size of the queue, the analysis being carried out, and the size of the file. When we ran these analyses ourselves, the “Make scaled coverage graph” job took 7 min and 12 s for the SRR10060911.bam as its file size is 1.83 GB, whereas SRR10060912.bam took only 5 min and 52 s, most likely due to its smaller file size of 455 MB. Larger files may take longer, for example, an 8.89 GB file took 38 min and 54 s to complete. Independent of BioViz *Connect*, the CyVerse infrastructure sends an email to users when jobs finish. When a job finishes, any files or folders it creates appear in the analyses folder in the user’s home directory, or in the same location as the input files, if those are stored in a location where the user has permission to modify or add to the folder. To quickly navigate to results, users can click the analysis name in the Analyses History, opening the folder where the output data files are stored.

Figure 4D shows sample App output, a visualization of the SR45A region with three scaled coverage graphs loaded from bigwig data files, a compact binary format for representing numeric values associated with base pairs in a genome map. Two heat-treated and one control sample are shown. The three coverage graphs have been configured to use the same y-axis scale, making it obvious that the heat treatment elevated SR45A gene expression, consistent with previously published reports. The image presents a clear visual argument in favor of this conclusion, and it also shows the user how much the expression level measurement varies across the gene body, something a single summary statistic cannot provide.

## Discussion

BioViz *Connect* introduces and demonstrates innovations in the field of science gateway development and research, while providing useful functionality for researchers seeking to understand and visualize genomic data. BioViz *Connect* enables users of the CyVerse science gateway to visualize genomic data files from their CyVerse accounts using Integrated Genome Browser, a desktop application. To our knowledge, BioViz *Connect* is the first and only resource that integrates remote CyVerse file storage and computational resources with a genome browser native to the local computer, achieving this cross-application communication *via* localhost REST endpoints.

We implemented BioViz *Connect* using the CyVerse Terrain API, a collection of remote REST endpoints that form a comprehensive computational interface to CyVerse resources. However, CyVerse and its Terrain API were not the first cloud system we considered. Our larger goal was to expand users’ experience of genome browsing by connecting the interactivity and speed of a native, desktop genome browser (such as IGB) with the vast resources of cloud-based, remote storage and computing systems, making it easier for users to store and share their data with others and also run compute-intensive visual analytics algorithms that would never be possible using just the user’s personal computer. To achieve this, we considered several commercial and public-sector systems, but selected CyVerse because of its focus on supporting scientific research, its free cost for users, and its early support for computational interfaces *via* APIs (Dooley et al., 2012).

At first, we proposed to use the CyVerse Agave API, which was well-documented and well-supported at the time. Since then, at least two other groups have published workflow management sites that use Agave, justifying our original choice (Wang et al., 2018; Hubbard et al., 2020). However, several months after launching our project, we discovered that Agave’s manipulation of user data conflicted with CyVerse’s own Discovery Environment interface, then a Web interface resembling a personal computer desktop. We also learned that Agave lacked support for HTTP range requests against data files, an essential feature from our perspective, and that this feature was unlikely to be added, as Agave’s maintainers were in the process of migrating to a new version to be called “Tapis.” Realizing our problem, they recommended we instead use Terrain, the API that powers the Discovery Environment interface. After consulting with developer teams working on Terrain and Discovery Environment, both based at University of Arizona, we decided to use Terrain.

We chose to use Integrated Genome Browser as the visualization component of BioViz *Connect* for several reasons. The first was that we wanted to demonstrate and explore a connection between cloud-based resources and a pre-existing, native, desktop application already in wide use, and IGB satisfied this requirement. The second major reason was convenience. As the core development group for IGB, we understand its architecture and capabilities, reducing our learning curve when connecting this local application to the cloud. IGB already contained a localhost REST interface that we could repurpose for BioViz *Connect*, an endpoint was first developed in the early 2000s to enable a connection between the Affymetrix NetAffx Web site and IGB. Since then, we used this same endpoint to implement IGB’s internal region and data bookmarking system. IGV, the only other native genome browser application in wide use, has a similar REST endpoint used to trigger loading of data files from the Galaxy Web site and others, but this endpoint lacks features such as the ability to specify track appearance. The third reason was that the IGB interface decouples navigation and data loading, thus making it easier for users to control when data are requested from the remote host. We surmised that this would make possible delays in data loading less onerous than for other browsers, such as IGV (Robinson et al., 2011), UCSC Genome Browser (Kent et al., 2002), Jbrowse (Buels et al., 2016), and Ensembl (Howe et al., 2021), all of which load data automatically when users navigate to a new region. However, since we first released BioViz *Connect*, the CyVerse development team have improved data throughput, making those tools’ design less problematic.

Our success in linking IGB to the cloud, along with the abovementioned improvement in CyVerse infrastructure, suggests an interesting next step for BioViz *Connect*: adding other genome browser systems to the interface. Anticipating this possibility, the first column in the BioViz *Connect* file browser table is labeled “Visualization Tools,” a generic heading that suggests adding other tools. Doing this would be valuable because although genome browsers often recapitulate each other’s features, all have capabilities unique to them, and users who prefer them. For example, IGB offers fast navigation through a genome, the ability to interact directly with data, access to shared data *via* IGB Quickload sites, and visual analytics functions called “Operations” that aid exploratory analysis. Unique features of the Broad Institute’s IGV include a sashimi plot view for detecting differential splicing (Katz et al., 2015) and a bisulfite sequencing view for understanding DNA methylation. The UCSC Genome Browser excels at offering a multitude of data sets in distinct tracks, while the Ensembl browser and associated informatics system famously support nearly every reference assembly known to science, including many plant genomes not supported by UCSC. And the Jalview system provides a host of features for examining the deep details of alignments, the heart of genomic analysis (Procter et al., 2021). BioViz *Connect* could make these systems easier to use and compare, allowing us to study how different approaches to visualization affect understanding.

To our knowledge, BioViz *Connect* is the first application developed using the Terrain API by a group outside the CyVerse development team. Because our work is open source, developed entirely in public, other groups can use our implementation as a guide or inspiration for their own work. BioViz *Connect* further demonstrates to the larger community of biologists, developers, and funders that modern, feature-rich REST interfaces to powerful computational resources stimulate and enable innovation and progress.

The scaled coverage graphs described in the use case scenario offer a useful, practical example of how remote resources can power interactive visual analytics on the desktop, an idea that has been explored in diverse fields and settings, but not often applied to genome visualization as was done here. The example we presented used a pre-existing algorithm, developed by others, but it shows how developers can harness a more powerful gateway system to develop and deploy all-new interactive genome data visualizations. Offloading compute-intensive visual analytics functions to science gateway systems will likely become more appealing and important as the size and complexity of genomic data continue to increase.

## Limitations and Ways to Overcome Them

However, at least two important technical limitations remain, providing opportunities for future work. The first technical limitation has to do with how data flows from the CyVerse back end data store and into the desktop genome browser application. Integrated Genome Browser as currently implemented can display data from users’ CyVerse accounts because the Terrain API can assign publicly accessible URLs to individual data files, which makes them available for visualization but exposes them to everyone on the internet. This problem of public accessibility could perhaps be addressed by adding password protection to these URLs, using Basic Authentication headers defined by the HTTP protocol. IGB already supports logging into password-protected Web servers, and so this solution would require little or no changes on the client side.

Another problem has to do with the data file formats themselves and how they can sometimes expose more information than anticipated. IGB, along with every other genome visualization system we are aware of, uses random access, indexed file formats to retrieve subsets of data corresponding to genomic regions. For example, BAM (binary alignment) files are typically large, impractical to download in their entirety. The data stored in these files are sorted by genomic location and therefore can be indexed by genomic location. When retrieving data for a desired genomic region, IGB and other programs use the BAM file’s index, stored separately in a smaller “bai” file, to look up the range of bytes where those data reside in the target file, and then read and process only the data for that region, ignoring the rest. This idea of mapping genomic coordinates to physical file coordinates has been in heavy use for decades, for as long as IGB has existed. Indeed, the original IGB development team at Affymetrix implemented one of the first indexed file formats, called “bar” for “binary array format”, used for storing and accessing data from Affymetrix genome tiling arrays, one of the first technologies invented to survey transcription across an entire genome in an unbiased way. However, in some situations, the index can sometimes serve as a genomic map, providing an overview of an entire dataset that could identify an individual. For example, as shown in (Pedersen et al., 2017), one can use the BAM index to detect chromosome abnormalities from whole genome sequencing data, exposing more information about a person or an experiment than anticipated.

The second technical limitation concerns how to flow data from remote sites, *via* a Web browser, into other programs running natively on the desktop, such as Integrated Genome Browser. Web browser development communities are constantly changing and improving their security models, essential to keeping users and their data safe in an increasingly adversarial and dangerous digital environment. Most Web pages are now loaded over encrypted channels, using HTTPS, the secure version of HTTP, and this includes BioViz *Connect*. This means that the JavaScript code responsible for interacting with IGB’s localhost endpoint is also loaded *via* HTTPS. However, when this code interacts with IGB *via* its localhost endpoint, it does so *via* unencrypted HTTP, because there is currently no robust way to support HTTPS for the localhost domain. The Chrome and Firefox browser allow BioViz *Connect* code to access the localhost IGB endpoint using HTTP because the communication channel is limited to the user’s own computer, presumed to be secure. The MacOS Safari Web browser does not allow it, however. This means that BioViz *Connect’s* “View in IGB” feature fails for Safari users. We handle this by advising the user to switch to a different browser on MacOS. This issue exemplifies a more general problem with connecting the desktop to the cloud. The methods used to communicate with remote computers are always changing, usually becoming more restrictive, which means that developers need to constantly test, revise, and update their software, more so perhaps than developers who create stand-alone, independent applications that rarely need to interoperate with anything other than the host computer’s operating system.

Architectures using Web-based REST APIs may help solve these problems. For example, CyVerse or BioViz *Connect* could add new endpoints that themselves support region-based retrieval of genomic data, as with the XML-based Distributed Annotation Service (Dowell et al., 2001; Jenkinson et al., 2008), the newer JSON-based University of Santa Cruz Genome Informatics REST interface (UCSC, 2021), or the BEACONS network API, which supports multiple layers of user authentication (https://beacon-project.io/). Rather than deliver data in new JSON or XML formats that would require modifying the client software, these new endpoints could simply stream the data in their native formats, requiring minimal or no change to the client software. Another way to achieve this would be to design APIs using the facade design pattern, in which an application translates an incompatible interface to a compatible one, expanding the range of clients able to access a resource. For example, developers could create a novel API that provides all the services required for accessing BAM files and their indexes, by creating and destroying secure URLs as users open and load data file resources during a session. Many variations are possible, and as cloud computing infrastructures become easier and cheaper to build upon, more bioinformatics groups will attempt even more daring and exciting innovations, amplifying their users’ ability to investigate biological systems.

Finally, we highlight aspects of the BioViz *Connect* interface and functionality that could be further developed to help users find useful tools and help developers find users for their tools. First, we note that the “View in IGB” button in the BioViz *Connect* table view occupies a column labeled “Visualization Tools,” a space where links to other visualization tools could also be added, based on the input data they accept. To make space for these other tools, we could replace the button with an IGB logo, and use tooltips to provide documentation or link to videos describing how to use the tools. Second, we could enhance BioViz *Connect* search capabilities to query MetaData tags or other file properties and attributes. Third, we could collaborate with the CyVerse team and other users to design and implement data registries, which data providers and users could use to publish, publicize, and locate data sets relevant to their work. As we hope the name suggests, BioViz *Connect* will connect researchers with data and tools, and will help tool developers connect with their intended audience, improving scientific practice for everyone.

## Data Availability

Publicly available datasets were analyzed in this study. This data can be found here: https://www.ncbi.nlm.nih.gov/sra/?term=PRJNA509437.

## Abbreviations

API, Application Programming Interface; CSS, Cascading Style Sheets; HTML, HyperText Markup Language; IGB, Integrated Genome Browser; REST, REpresentational State Transfer.
